# Supplementary material for: Rapid turnover of life-cycle-related genes in the brown algae
Source: Genome Biol. 2019 Feb 14;20:35. doi: 10.1186/s13059-019-1630-6 (PMC6374913; doi:10.1186/s13059-019-1630-6)
Supplement: Supplementary file 3 — Protein alignments, corresponding Gblocks html file and CDS sequences for the lineage conserved gametophyte-biased genes. (ZIP 2109 kb) [file 13059_2019_1630_MOESM3_ESM.zip › Con_GA_alignments/Ec-01_007050.aa_ali.fasta-gb.htm]

Ec-01\_007050.aa.fasta.trans.aa\_ali.fasta


## Gblocks 0.91b Results

Processed file: **Ec-01\_007050.aa.fasta.trans.aa\_ali.fasta**  
Number of sequences: **2**  
Alignment assumed to be: **Protein**  
New number of positions: **392** (selected positions are underlined in blue)

```
                         10        20        30        40        50        60
                 =========+=========+=========+=========+=========+=========+
Ec-01_007050     MRSLLHALPAALLSVLGGTSGVASQIQPAVVESFAHSALLSVRVVDGEPNDAGDHEAMAV
MSTRG.43671.1    ------------------------------------------------------------
                                                                             


                         70        80        90       100       110       120
                 =========+=========+=========+=========+=========+=========+
Ec-01_007050     VVSTITEMDSDGKPVGVEGSPLFFDSDNISVSSTRRSGVYQDGLDAEEVSSVATTTGTAT
MSTRG.43671.1    ------------------------------------------------------------
                                                                             


                        130       140       150       160       170       180
                 =========+=========+=========+=========+=========+=========+
Ec-01_007050     TVSLVATNYLGDGNISTFEPSTLVEVGNETVRLDVRIRDWSFCTPFGRDGGRRCMAEPQD
MSTRG.43671.1    ------------------------------------------------------------
                                                                             


                        190       200       210       220       230       240
                 =========+=========+=========+=========+=========+=========+
Ec-01_007050     IFLQNEGALPLPPAPPPPHTLLIPRCDGETVATFRFVCDPRLVECADVVSVKYYLGEADQ
MSTRG.43671.1    ------------------------------------------------------------
                                                                             


                        250       260       270       280       290       300
                 =========+=========+=========+=========+=========+=========+
Ec-01_007050     DPGQAPNTATIGVARGSGENGTSTSSSGALEEFWGTGVYERNLTRAEVGSFLNVSSLTPG
MSTRG.43671.1    -----------------------------MEAFGSVGVYEHNLTNVEVGGFLNVSSLTPG
                                               ##############################


                        310       320       330       340       350       360
                 =========+=========+=========+=========+=========+=========+
Ec-01_007050     LDYVFWSVWEHGNGSAPESNSRDFDLPRVQYWQCPGTTIKRNFEVGTSLSVSLVVNGSTA
MSTRG.43671.1    LDYVFWSVWEHGNGSVPESNSRDFDLSRSQFWQCPGTTIERNFEVGTSLSVSLLVNGSSA
                 ############################################################


                        370       380       390       400       410       420
                 =========+=========+=========+=========+=========+=========+
Ec-01_007050     LVPLCGNSVSASSPDSSNTGPTSSAASSSSTTSGSSSCQKIGTDGVATETSGPSRRLSDP
MSTRG.43671.1    LDPFCEEFSPESS--SSEFSSCRQASSSASYSDG----------ATASAVANDRRRLYGT
                 #############  ###################            ##############


                        430       440       450       460       470       480
                 =========+=========+=========+=========+=========+=========+
Ec-01_007050     GDESHPSPALPARQG--RGYRLLQEDQGS-SASLDQTEGGAGWAWDSDGPIFGLGELSRI
MSTRG.43671.1    SDDSRSRSFLSGRRGRGRGHRLLQEDEDTHNATRDQAAGDGGWAWDSDGPRFGLGERSKI
                 ###############                #############################


                        490       500       510       520       530       540
                 =========+=========+=========+=========+=========+=========+
Ec-01_007050     RLSSHVLLLGDDDNDSSDYEEWTPLRNTSSWVATAAAGATENEGGGGEGRAGVITASLEP
MSTRG.43671.1    RLSSHVLLLDGSDGNT---GVWAPLRNTSSASAVEVSEASDEE---GEGRAGVITASLDS
                 #############        ######################   ##############


                        550       560       570       580       590       600
                 =========+=========+=========+=========+=========+=========+
Ec-01_007050     FDSQAVFSALLFFSKVEVIQEESDKGVEAATWVAIAFGFLLLWLLGCYGVRLAKHRHAAK
MSTRG.43671.1    FEATAVFSTLLYFSSVESAEDSSGSGDEAATWVAIAFAFFLVGLLGWCSIRLSKKSHAAK
                 ############################################################


                        610       620       630       640       650       660
                 =========+=========+=========+=========+=========+=========+
Ec-01_007050     TWPSAVARAAARSHGGLFDDDASSFGSPTSSEADSSMGGVKELQIFPMMMKAEF---KS-
MSTRG.43671.1    TWPTEVARAAARNKFGFDPGKESTFGSPSSSDGGTSMGGVKELQIFPVMMAPRTKYSRSN
                 ##################################################          


                        670       680       690       700       710       720
                 =========+=========+=========+=========+=========+=========+
Ec-01_007050     -KLEFSDRSDAVRSTSAEEDEQSICAPKAPGWTTPGSGSLNCIHDQNSSSNNNKNNNADS
MSTRG.43671.1    YDVEGTRKSATATSKSSEGGAAPLRTPPAPGWTTPGSGSS---KHTTLNSHGNDDHDHDG
                    ####################################          ###########


                        730       740       750       760
                 =========+=========+=========+=========+
Ec-01_007050     NRNVDNGNMASEVFPSPPPFHYRVPAEAFPLTDAMLELPV
MSTRG.43671.1    KNKFSNDEKDDRC---------------------SSSSSX
                 ######
```

```
Parameters used
Minimum Number Of Sequences For A Conserved Position: 2
Minimum Number Of Sequences For A Flanking Position: 2
Maximum Number Of Contiguous Nonconserved Positions: 8
Minimum Length Of A Block: 10
Allowed Gap Positions: None
Use Similarity Matrices: Yes
```

```
Flank positions of the 8 selected block(s)
Flanks: [271  373]  [376  394]  [407  435]  [452  493]  [502  523]  [527  650]  [664  699]  [710  726]  

New number of positions in Ec-01_007050.aa.fasta.trans.aa_ali.fasta-gb:  392  (51% of the original 760 positions)
```
